# Sprague Dawley Rats Gaining Weight on a High Energy Diet Exhibit Damage to Taste Tissue Even after Return to a Healthy Diet

**DOI:** 10.3390/nu13093062

**Published:** 2021-08-31

**Authors:** Fiona Harnischfeger, Flynn O’Connell, Michael Weiss, Brandon Axelrod, Andras Hajnal, Krzysztof Czaja, Patricia M. Di Lorenzo, Robin Dando

**Affiliations:** 1 Department of Food Science, Cornell University, Ithaca, NY 14850, USA; fh268@cornell.edu (F.H.); bha36@cornell.edu (B.A.); 2 Department of Psychology, Binghamton University, Box 6000, Binghamton, NY 13902, USA; foconne1@binghamton.edu (F.O.); weissm@binghamton.edu (M.W.); diloren@binghamton.edu (P.M.D.L.); 3 Department of Neural and Behavioral Sciences, College of Medicine, The Pennsylvania State University, Hershey, PA 17033, USA; ahajnal@psu.edu; 4 Department of Veterinary Biosciences and Diagnostic Imaging, University of Georgia, Athens, GA 30602, USA; czajak@uga.edu

**Keywords:** taste buds, obesity, diet

## Abstract

Many reports detail taste dysfunction in humans and animals with obesity. For example, mice consuming an obesogenic diet for a short period have fewer taste buds than their lean littermates. Further, rats with diet-induced obesity (DIO) show blunted electrophysiological responses to taste in the brainstem. Here, we studied the effects of high energy diet (HED)-induced peripheral taste damage in rats, and whether this deficiency could be reversed by returning to a regular chow diet. Separate groups of rats consumed a standard chow diet (Chow), a HED for 10 weeks followed by a return to chow (HED/chow), or a HED for 10 weeks followed by a restricted HED that was isocaloric with consumption by the HED/chow group (HED/isocal). Fungiform taste papilla (FP) and circumvallate taste bud abundance were quantified several months after HED groups switched diets. Results showed that both HED/chow and HED/isocal rats had significantly fewer FP and lower CV taste bud abundance than control rats fed only chow. Neutrophil infiltration into taste tissues was also quantified, but did not vary with treatment on this timeline. Finally, the number of cells undergoing programmed cell death, measured with caspase-3 staining, inversely correlated with taste bud counts, suggesting taste buds may be lost to apoptosis as a potential mechanism for the taste dysfunction observed in obesity. Collectively, these data show that DIO has lasting deleterious effects on the peripheral taste system, despite a change from a HED to a healthy diet, underscoring the idea that obesity rather than diet predicts damage to the taste system.

## 1. Introduction

From 1975 the prevalence of obesity has tripled across the world [1]. With this growing obesity epidemic, food intake is posited as the primary driver of obesity [2]. Per capita energy intake and the size of food portions, especially foods with low levels of nutrients, have been steadily rising [3,4,5], with fat and sugar increasingly available in the modern food system [6,7,8,9]. The habitual excess intake of fat and sugar leads to a chronic positive energy balance, which in turn causes weight gain [10,11,12,13]. In developed countries this problem is more acute, as more foods high in saturated fat and added sugar are more regularly consumed [14,15,16,17].

As our primary driver of food choice is taste [18,19,20,21], understanding how the taste system is affected with obesity may shed light on why obesity is so difficult to treat. Sensory studies on panelists with obesity have shown a dampened sense of taste in many designs [22,23,24,25,26,27,28,29,30,31,32]. Alongside sensory changes observed in obese subjects, physiological changes to taste have also been identified in mice and humans.

Humans and other mammals perceive the taste of foods through activation of taste receptor cells, which are collected in structures called taste buds. Three types of papillae on the tongue house the taste buds. The fungiform papillae (FP) are located on the anterior 2/3 of the tongue’s dorsal surface and each contain several taste buds. Foliate papillae are collected in folds on the posterior lateral surface of the tongue while circumvallate (CV) papillae are large structures located at the rear of the tongue. Humans have a number of CV papillae, while rodents have a single CV at the back of the tongue. Both foliate and CV papillae house a large number of taste buds. Taste receptor cells are innervated by three cranial nerves, VII, IX and X, that project to gustatory nuclei in the brain [33,34,35,36,37].

Both taste buds and central gustatory nuclei have been shown to be altered in subjects with obesity. In adults and children with obesity, the density of fungiform papillae negatively correlated with adiposity [29,38]. Consistent with these findings, a 4-year longitudinal study on college students testing FP density change revealed a correlation with changes in adiposity [39]. In rodents, diet-induced obese mice were found to have fewer taste buds than chow-fed, lean littermate mice [40]. This reduction in taste bud abundance was found to be linked to inflammation. In obese rodents, molecular evidence shows a decreased response to fat and sweet stimuli using calcium signaling as well as a decreased expression level of taste markers, including lower mRNA expression for the taste receptor type 1 member 3 (T1R3) involved in sweet and umami taste detection [41,42,43,44,45] Recently, evidence has emerged from single-cell RNA sequencing experiments of human fungiform taste papillae that showed altered gene expression, specifically reduced expression of the type II taste cell marker PLCβ2, and increased expression of genes associated with inflammation [46]. Further links between taste and inflammation are reviewed by Goodman & Dando [47]. In the first nucleus in the central gustatory pathway in rats, the nucleus of the solitary tract, electrophysiological responses to taste stimuli are blunted, with lower magnitude, longer latency and shorter duration, compared with cells in lean rats [48]. Similar blunted taste-responsiveness was observed in the pontine parabrachial nucleus, the second central taste relay, in obese rats [49].

In this study, we hypothesized that rats would lose taste buds with obesity, and that a return to standard chow would at least partially reverse the effects of obesity on taste buds, when compared to the effects of a restricted, high-energy diet (HED). These effects include altered abundance of taste buds, measured in both the fungiform and circumvallate regions, and density of neutrophils in taste tissues, measured via the marker myeloperoxidase (MPO). Neutrophils are effectors of the inflammatory response, were demonstrated 100 years ago to infiltrate taste buds after injury [50], and result in deficiencies in taste function when recruited to injured or healthy taste buds [51,52]. In this manner, we aimed to gauge the effectiveness of a dietary shift on mitigating the damage to taste from diet-induced obesity.

## 2. Materials and Methods

### 2.1. Animals

Male Sprague-Dawley rats (*n =* 23; Taconic Labs, Inc., Germantown, NY, USA) were kept on a 12-h light/dark cycle at Binghamton University and approved by Binghamton University IACUC under 795–18. Animal treatments were performed at Binghamton University, with all tissue work and counting taking place blinded at Cornell University, to avoid bias. Rats were 14 weeks old at the beginning of the experiment and were single-housed. Animals were divided into three groups: One group, “Chow” (*n =* 9), was fed standard chow (PicoLab Diet 5L0D, 13% kCal fat, 58% kCal carbohydrates, 29% kCal protein, PicoLab, St.Louis, MO, USA) *ad libitum* for the entire experiment. A second group, “HED/chow” (*n* = 7), was fed a HED (Research Diets D12451, 45% kCal fat, 35% kCal carbohydrate, 20% kCal protein, Research Diets, New Brunswick, NJ, USA) *ad libitum* for 10 weeks and then switched to standard chow *ad libitum* for the remainder of the experiment. The final group, “HED/isocal” (*n* = 7), was fed a HED *ad libitum* for 10 weeks, and then fed the same HED isocalorically matched to the caloric consumption of the HED/Chow group. Rats were fed once a day when restricted, and ad-libitum when not. Rats in the HED/chow were weight-matched to counterparts in the HED/isocal group. The rats spent 10 weeks on either chow or HED followed by 38.8 (+/−1.8) weeks on either chow or HED that was isocaloric to the chow intake of the other HED-treated group. Food intake was measured daily in the HED/chow rats and each rat in the HED/isocal group was given a portion of HED food that was isocaloric to what their partner in the HED/chow group had eaten the day before. Food intake in both groups was measured daily and compared for 8 of the 10 weeks post-HED treatment. Occasionally the portion of food in the HED/isocal group may not have been 100% completely consumed due to hoarding behavior displayed by the rats. Body weights were measured every weekday for the entire post-treatment period. Three of the 6 rats in the HED/isocal group were put back on a chow diet shortly before they were sacrificed; statistical testing revealed no significant difference in any measures (MPO/caspase/CV or fungiform taste bud counts) between the groups, so the rats were pooled for analysis.

Rats were sacrificed with 100–180 mg/kg Sodium Pentobarbital, tongues were excised, placed in 4% PFA (Fisher Scientific, Hampton, NH, USA) /PBS (Fisher Chemicals, Hampton, NH, USA) for 1 ½ h, washed with PBS 3x for 20 min, cryoprotected in sucrose overnight (Fisher Chemicals, Hampton, NH, USA), embedded in OCT (Fisher Scientific, Hampton, NH, USA), and frozen at −80 °C. Not all analyses were performed on all samples, due to occasional tissue processing issues.

### 2.2. Dual-Energy X-ray Absorptiometry Scans

Dual-energy X-ray absorptiometry (DXA) scans were taken approximately every 2–3 wks throughout the experiment as well as before sacrificing to determine body composition. Before scanning, animals were sedated Dormitol (Medetomidine HCl, 0.1 mg/kg, SC, Pfizer, Inc., New York, NY, USA) and were revived with Antisedan (Atipamezole, 0.1 mg/kg, IP, Pfizer, Inc., New York, NY, USA).

### 2.3. Fungiform Papillae Staining and Counting

Tongues were extracted, cryoprotected with sucrose, and frozen for transport. After thawing, fungiform papillae were dissected and tongues were stained with 0.025% methyl blue PBS (VWR, Radnor, PA, USA) for 30 seconds and then rinsed in diH20 for 1 ½ h. Pictures of the stained tongues were taken with an Olympus SZ61 dissection scope (Olympus Optical, Tokyo, JP) in combination with a Lumenera Infinity 1080p60 HD microscopy camera (Lumenera, Ottawa, CA, USA). To count fungiform papillae a 3 mm by 3 mm box was aligned with the tongue’s midline 4 mm from the tip of the tongue with a second box mirrored across the midline. The number of fungiform papillae was counted via methyl blue staining in both boxes and averaged, with data reported as papillae/mm^2^. Staining, counting and analysis were performed by blinded personnel.

### 2.4. Immunohistochemical Staining and Counting

Tongues were extracted, tissue regions were dissected from tongues, cryoprotected with sucrose, and frozen for transport. After thawing, CV tissue regions were dissected from tongues and placed in OCT medium as above. Circumvallate tissues were cryosectioned at 10 um thickness, washed in PBS (VWR, Radnor, PA, USA), and incubated in 1% triton (MilliporeSigma, Burlington, MA, USA). Tissue sections stained with primaries: 1:500 polyclonal Goat GNAT3 OAEB00418 (α-gustducin) from Aviva Systems Biology (San Diego, CA, USA); 1:125 polyclonal Rabbit Caspase-3 AF835 from R&D systems (Minneapolis, MN, USA) and incubated with 4% bovine serum albumin (BSA) (Amresco, Solo, OH, USA), 4% donkey serum (Equitech-Bio, Kerrville, TX, USA), and 0.3% triton (MilliporeSigma, Burlington, MA, USA). Tissue sections stained with 1:125 polyclonal Goat MPO AF3667 (labelling neutrophils) from R&D systems (Minneapolis, MN, USA) were blocked for 2 h at room temperature with 2% BSA (Amresco, Solo, OH, USA), 2% donkey serum (Equitech-Bio, Kerrville, TX, USA), and 0.3% triton (MilliporeSigma, Burlington, MA, USA). After incubation with Alexa Fluor donkey anti-Goat or anti-Rabbit secondary (Invitrogen, Carlsbad, CA, USA) at room temperature for 2 h, sections were washed 3× for 20 min in PBS (VWR, Radnor, PA, USA), and placed on a coverslip with DAPI staining medium (Fluoromount-G, Southern Biotech, Birmingham, AL, USA). Tissue sections were imaged using an Olympus IX-71 inverted scope and Hammatsu Orca Flash 4.0 camera (Hamamatsu Photonics, Hamamatsu City, Japan). Taste buds, Caspase-3 positive cells and neutrophils in tissue samples were counted using ImageJ (NIH, Bethesda, MD, USA). Immunohistochemistry, counting and analysis were performed by blinded personnel.

### 2.5. Statistical Analysis

Statistical analysis was performed with GraphPad Prism 7 (GraphPad Prism, San Diego, CA, USA). Groups were compared using non-parametric Kruskal-Wallis tests due to sample sizes, with statistical significance assumed at *p* < 0.05.

## 3. Results

**Figure 1:**
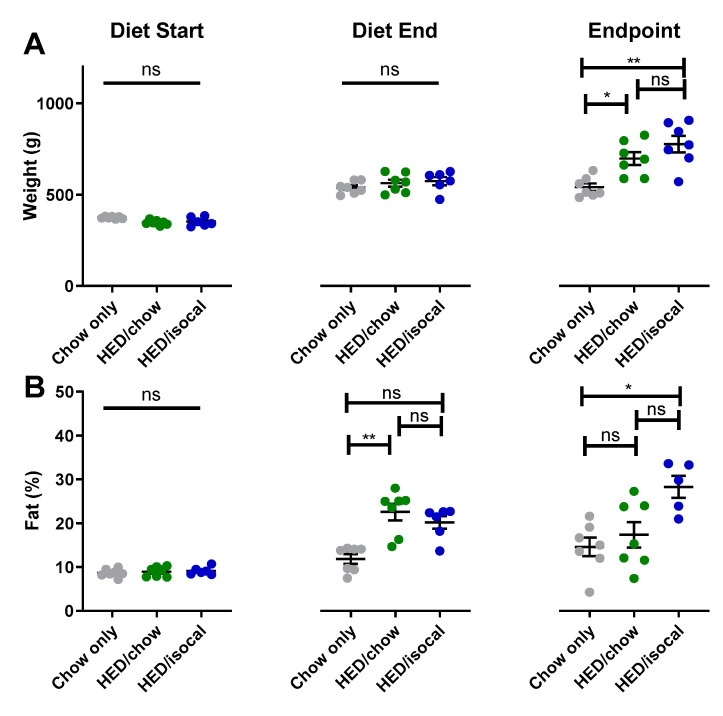
(**A**) Weight (g) of chow-only (grey, *n =* 7), HED/chow (green, *n =* 7) and HED/isocal (blue, *n =* 7) rats at the beginning of the first diet, after 10 weeks on HED or chow, and the endpoint/ day of tissue collection. (**B**) Percent body fat of chow-only (grey, *n =* 7), HED/chow (green, *n =* 7), and HED/isocal (blue, *n =* 6) rats at the beginning of the first diet, after 10 weeks on HED or chow, and the endpoint/day of tissue collection. Stars represent statistical significance, where * = *p* < 0.05; ** = *p* < 0.01; ns denotes non significant. Bars represent means plus/minus SEM. HED = high energy diet; isocal = isocalorically fed.

When diets for 2 groups consuming HED were switched, rats had significantly higher body fat percentage, although were not statistically heavier, likely due to a slightly smaller body size at the start of the study (Supplemental Figure S1). At the endpoint of the experiment, both groups of rats treated with HED were heavier than control chow-fed rats (Figure 1A, HED/chow *p =* 0.042; HED/isocal *p =* 0.002), with HED/isocal rats also having more body fat (Figure 1B, *p =* 0.023). Food intake was also compared in the period post- HED treatment. HED/chow fed rats ate an average of 9.8% more chow than chow-only fed rats across this period (as can be said by extension of their isocalorically fed HED/isocal counterparts), representing a daily average of 89.0 calories compared to 81.0 calories during the same time period, and at the same age (*p* < 0.001).

**Figure 2:**
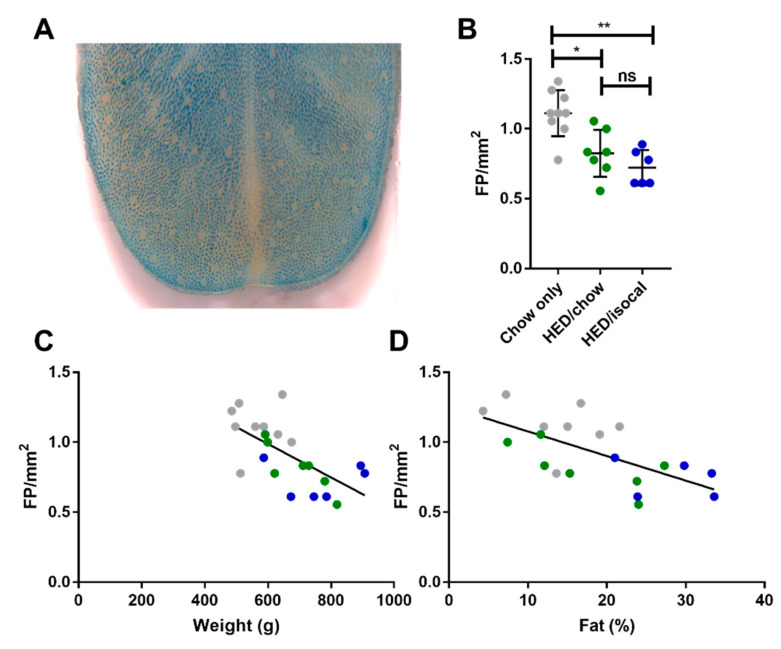
(**A**) Representative image of anterior tongue, with fungiform papillae the pink regions not taking up methyl blue dye. (**B**) Fungiform Papillae (FP) density in rats consuming chow-only (grey, *n =* 9), high-energy diet (HED) then chow (green, *n =* 7), and HED then HED isocaloric (blue, *n =* 6). (**C**) Fungiform papillae (FP) per mm2 (y-axis) versus Weight (g) (x-axis). Chow-only (grey, *n =* 9), HED then chow (green, *n =* 7), and HED then HED isocaloric (blue, *n =* 6). Pearson’s *r* = −0.646, *p =* 0.0012. (**D**) Fungiform papillae (FP) per mm2 (y-axis) versus Body Fat (%) analyzed by DXA scan (x-axis). Chow-only (grey, *n =* 9), HED then chow (green, *n =* 7), and HED then HED isocaloric (blue, *n =* 6). Pearson’s *r* = −0.655, *p =* 0.002. ns denotes non significant; * denotes *p* < 0.05; ** denotes *p* < 0.01.

Rats consuming chow-only had more fungiform papillae than those who consumed the HED, whether after being switched to chow (*p =* 0.037), or continued to be fed a HED isocalorically (*p =* 0.005). Even after 4 months consuming a chow diet, fungiform papillae did not recover to the level of chow fed counterparts. HED/chow and HED/isocal fed rats’ fungiform papilla density did not differ significantly (*p* > 0.999) (Figure 2).

**Figure 3:**
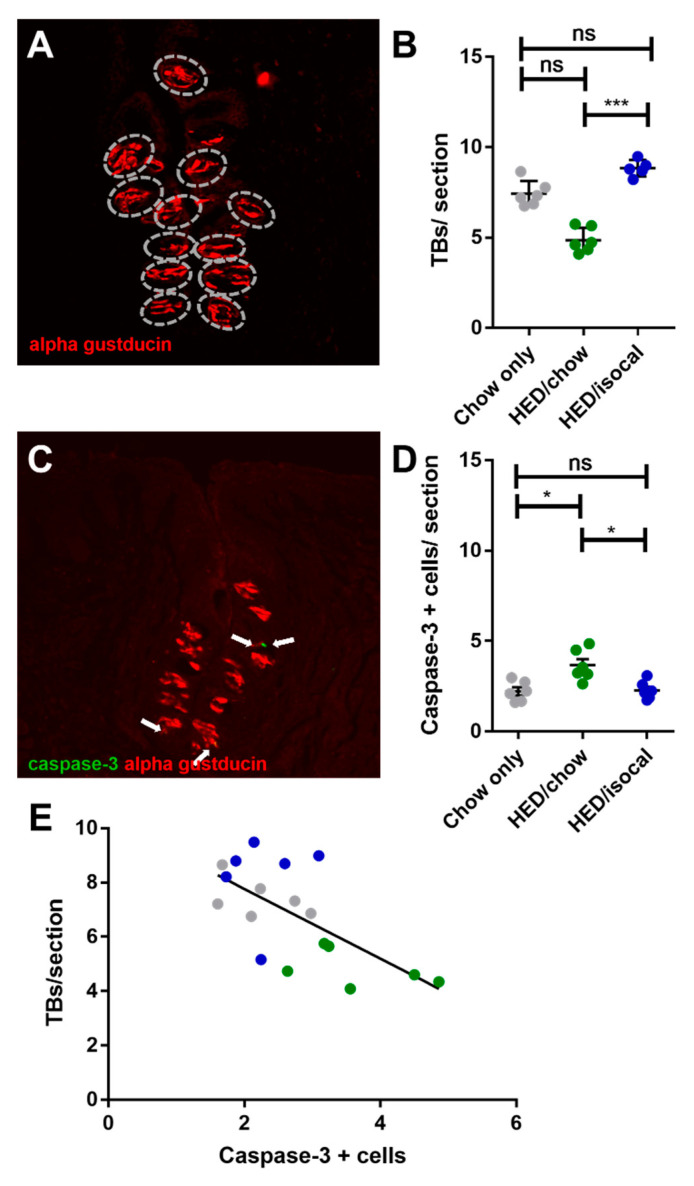
(**A**) Representative image of circumvallate papilla with α-gustducin (red) staining higlighting taste buds. (**B**) Number of taste buds (TBs) per CV section for all treatment groups. Chow-only (grey, *n =* 6), high-energy diet (HED) then chow (green, *n =* 7), and HED then HED isocaloric (blue, *n =* 6). Overall *p =* 0.029. Y-axis shows taste buds per section. (**C**) Representative image of circumvallate papilla showing caspase-3 (green), α-gustducin (red). (**D**) Caspase-3 positive cells per CV section for all treatment groups. Chow-only (grey, *n =* 6), HED then chow (green, *n =* 7), and HED then HED isocaloric (blue, *n =* 6). Bars represent means plus/minus SEM. Overall *p =* 0.001. (**E**) Number of taste buds/ section (y-axis), caspase + cells (x-axis). Chow-only (grey, *n =* 6), HED then chow (green, *n =* 7), and HED then HED isocaloric (blue, *n =* 6). Pearson’s *r* = −0.662, *p =* 0.003. ns denotes non significant; * denotes *p* < 0.05; *** denotes *p* < 0.001.

The number of FP across the 3 groups correlated negatively with both weight (Figure 2C, *r* = −0.646; *p =* 0.001) and with body fat (Figure 2D, Pearson’s *r* = −0.655; *p =* 0.002). Chow-only fed rats had more FP in the anterior region of the tongue versus rats fed a HED, however no alteration in CV taste buds, based on α-gustducin staining, was recorded between chow fed rats and either dietary treatment group. Post-hoc comparisons revealed a difference between HED/chow and HED/isocal rats (*p =* 0.001), with those consuming chow after HED having fewer taste buds than those maintained on restricted HED (Figure 3A,B).

**Figure 4:**
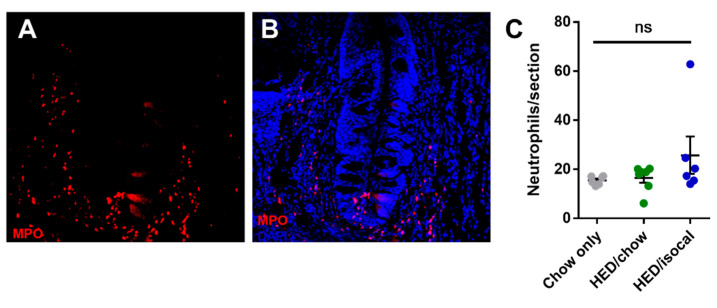
(**A**) Representative image of circumvallate papilla showing MPO staining (red). (**B**) Representative image of circumvallate papilla showing MPO staining (red), Dapi (blue) (**C**) MPO positive cells per CV trench for all treatment groups. Chow-only (grey, *n =* 6), high-energy diet (HED) then chow (green, *n =* 7), and HED then HED isocaloric (blue, *n =* 6). Bars represent means plus/minus SEM. Overall *p =* 0.141. ns denotes non significant.

As apoptosis was implicated in earlier work on obesity-driven taste bud loss and diabetes [40,53], CV taste sections were also stained for caspase-3. Rats fed HED/chow had a higher number of caspase-positive cells than chow-fed controls (*p =* 0.010) and HED/isocal-fed rats (*p =* 0.022) (Figure 3C,D). Moreover, the number of CV taste buds in all samples was inversely related to caspase 3-positive cells (Figure 3E, Pearson’s *r* = −0.662, *p =* 0.003). One outlier that was more than two standard deviations from the mean number of caspase positive cells was removed. Across groups there was no significant difference in the number of MPO-positive cells (*p =* 0.141), marking neutrophils, in the CV region (Figure 4).

## 4. Discussion

A relatively brief history of HED consumption was associated with long term changes in food intake, body weight and body fat, as well as possibly permanent changes in taste anatomy, regardless of changes to diet. At the conclusion of the experiment, rats in the HED/chow group and the HED/isocal group were significantly heavier than their chow-fed counterparts (Figure 1). Moreover, HED/isocal rats had a higher percentage of body fat compared to chow-only rats, with HED/chow rats having slightly (but not significantly) higher body fat percentage than chow-only rats. Previous work suggests that rats exposed to 2 weeks of a HED consumed significantly more calories than their chow-fed counterparts per meal [54]. Indeed, during the 8 weeks when intake was measured during the experiment, HED/chow rats ate significantly more than ad lib chow fed rats (9.8%).

The observed increase in overall food intake that was seen in the HED/Chow and HED/isocal groups may be the result of alterations to reward mechanisms induced by the HED. In human studies, circuits involved in reward and motivational salience have reduced response in obese patients [55,56,57]. Finally, HED in this study and in previous studies [39,40] has been shown to reduce the abundance of taste buds on the tongue, which speculatively may either cause or exacerbate reduced reward in the brain, and lead to hyperphagia as a compensatory mechanism for this reduced sensory input. HED-induced obesity in rats also blunts neuronal signals in the NTS [48]. A blunted neuronal response, or changes in reward mechanisms may persist after the dietary switch in our experiments and induce rats to continue to eat more after the switch from ad-libitum HED.

### 4.1. Fewer Fungiform Papillae in Rats Consuming HED Correlated Negatively with Weight and Body Fat

Rats fed a HED had significantly fewer FP compared to the leaner rats fed standard chow (Figure 2), with FP counts correlating negatively with both body weight and body fat (Figure 2). Previous studies have shown that FP density negatively correlates with adiposity in adults and children [29,38]. More recently, it was demonstrated that mice show a similar negative correlation between weight and number of FP, and in a longitudinal study of college students across 4 years showed a similar correlation between adiposity change and FP density change [39]. Thus, in rats, as in mice and humans, obesity is related to a reduction in the abundance of FP. While we do not directly enumerate fungiform taste buds, just the papillae in which they reside, we strongly suspect that a papilla density about half that of control rats (as seen in some of the HED/isocal rats) would result in a deficiency in the ability to taste in these animals. In the circumvallate taste field, HED/isocal rats seem to show an improved taste phenotype when compared to those switched to ad-lib chow after HED. This suggests there may be more to learn concerning the intersection between taste, obesity and diet.

The divergent trends observed in the FP compared to the CV papillae could have a variety of explanations. Anatomically, the two are located on different regions of the tongue and are innervated by different nerves. The chorda tympani branch of the facial nerve innervates the FP, while the glossopharyngeal nerve innervates the CV [58,59]. Previously, cross-regenerating those nerves alters taste perception [60]. It is possible that the regenerative capacity of these regions differs, or that the effects of HED may depend on differing vulnerability of these nerves to HED, leading to disparate effects with taste field. Nonetheless, we acknowledge that we have no data suggesting any changes in taste bud innervation produced by a HED. This result also raises the possibility that responses to basic tastes may be affected differentially after a HED, if indeed more bitter taste receptors are present in the CV than in fungiform taste buds.

### 4.2. Seemingly Superior Recovery of Taste Phenotype When Switching to a Restricted HED Regimen, versus Switching to an Ad Libitum Chow Diet

In a previous study, mice fed a HED alongside those consuming the HED plus a pharmacological agent to preclude weight gain still exhibited behavioral deficiencies in taste function [61], suggesting that a HED alone may be sufficient to damage taste buds as we hypothesize behavioral deficiencies and number of taste cells are linked. In fact, we saw little improvement in the abundance of taste cells after switching to a chow diet in our rats. While no loss in taste buds was reported in this study, taste bud abundance itself was not quantified, only taste cells per bud. Reducing the number of calories via isocaloric pair-feeding of C57BL/6J mice with a HED (58% fat), compared to a chow diet (11% fat) attenuated the development of obesity and type II diabetes seen in ad libitum HED-fed mice, but importantly did not completely ameliorate these effects [62]. In this study, we thus hypothesized that HED/isocal-treated rats would have fewer taste buds than chow-only or HED/chow-fed rats, which in fact was not the case. One study comparing weight loss in mice initially on a HED then switched to either a chow diet (10% fat) or a restricted HED (40.2%), showed that both a switch to chow and a high-fat 70% calorie-restricted diet induced weight loss. Interestingly however, they found that HED-restricted mice had a larger reduction of WAT inflammation, as measured by macrophage infiltration, and increase in mitochondrial carbohydrate metabolism [63]. The results of this study suggest that a restricted HED is superior to an ad libitum chow diet in controlling inflammation. One additional factor to note is that rats fed a restricted diet may become inadvertently fed in a time restricted fashion, akin to intermittent fasting, as observed in mice [63]. This can have a positive effect on metabolic disease, without reducing caloric intake [64]. Adipose tissue inflammation and fibrosis in a HED (43% fat) was ameliorated by 3 nonconsecutive days/week fast for 24 h [65]. In our experiments, a superior taste phenotype seemed to be evident in rats on the isocaloric HED regimen versus those consuming chow after the HED, at least in measures of taste bud counts, and of apoptosis, which is elevated in a pro-inflammatory condition.

### 4.3. More Cells in Taste Regions Undergoing Apoptosis in HED/Chow-Fed Rats

Caspases are subdivided into initiator caspases (caspase-2, -8, -9, and -10) and effector caspases (caspase-3, -6, and -7). The former act as triggers, while the latter execute apoptosis by acting directly on specific cellular substrates [66]. One mechanism that could explain a loss of taste buds in the HED/chow-fed rats is an increase in the number of cells undergoing programmed cell death, as marked by caspase-3, the primary executioner caspase of the apoptotic cascade (Figure 3). The abundance of taste buds in the rats exhibited a negative relationship with caspase-3-positive cells (Figure 3), consistent with fewer taste buds in rats coinciding with more apoptosis within taste regions.

## 5. Conclusions

Here we show Sprague-Dawley rats fed a high energy diet have fewer fungiform papillae than chow-fed control rats. Further, damage to gustatory anatomy is not reversed by switching to a reduced amount of HED, or back to regular lab chow. This implies that obesity-induced damage to the peripheral taste system may persist even after weight gain is resolved. An increase in cells undergoing apoptosis in the CV presents a potential mechanism for a loss of taste buds. One limitation of our study is the lack of a treatment group consuming HED for the duration of the study, made difficult due to the long timescale of experiments, and may be an area for future research. Our data suggest that a history of a HED, despite a long interval of a normal chow diet afterward, compromises the taste buds.

## Supplementary Materials

The following are available online at https://www.mdpi.com/article/10.3390/nu13093062/s1, Supplemental Figure S1. A: Weight (g) of chow-only (grey) versus high-energy diet (HED) consuming rats grouped (green) at the beginning of the first diet, and after 10 weeks on HED or chow. B: Percent body fat of chow-only (grey), and HED consuming rats pooled (green), at the beginning of the first diet, and after 10 weeks on HED or chow. Stars represent statistical significance, where * = *p* < 0.05; ** = *p* < 0.01; *** = *p* < 0.001. Bars represent means plus/minus SEM.
