# Supplementary material for: Sprague Dawley Rats Gaining Weight on a High Energy Diet Exhibit Damage to Taste Tissue Even after Return to a Healthy Diet
Source: Nutrients. 2021 Aug 31;13(9):3062. doi: 10.3390/nu13093062 (PMC8465157; doi:10.3390/nu13093062)

Supplemental Figure S1. A: Weight (g) of chow-only (grey) versus high-energy diet (HED) consuming rats grouped (green) at the beginning of the first diet, and after 10 weeks on HED or chow. B: Percent body fat of chow-only (grey), and HED consuming rats pooled (green), at the beginning of the first diet, and after 10 weeks on HED or chow. Stars represent statistical significance, where * = $p < 0.05$; ** = $p < 0.01$; *** = $p < 0.001$. Bars represent means plus/minus SEM.

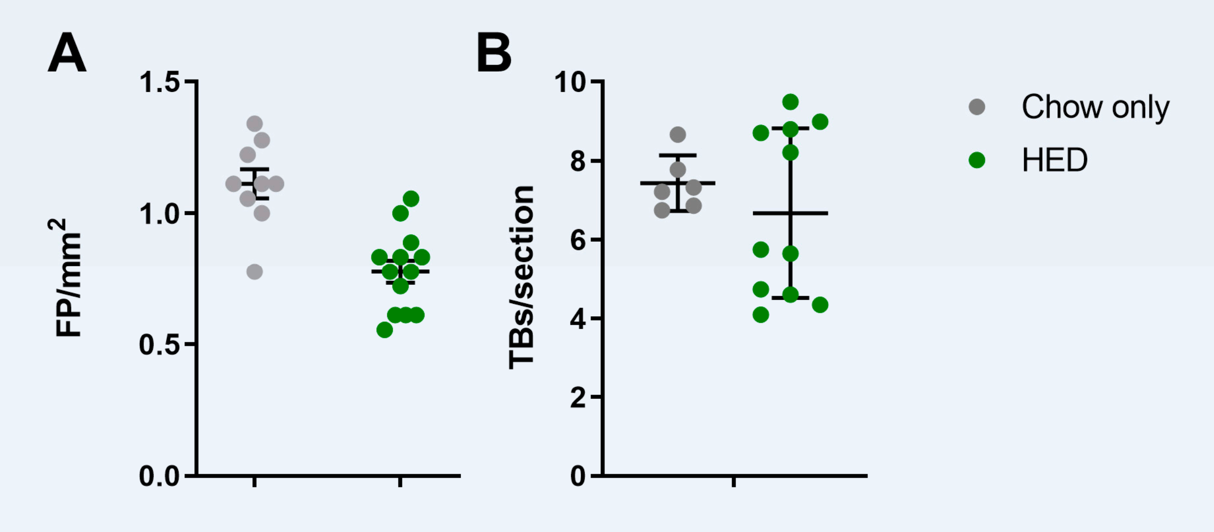

Supplement: Supplementary file 1 [file nutrients-13-03062-s001.zip › nutrients-1323495-supplementary.pdf]
